# Plant and Microbial Approaches as Green Methods for the Synthesis of Nanomaterials: Synthesis, Applications, and Future Perspectives

**DOI:** 10.3390/molecules28010463

**Published:** 2023-01-03

**Authors:** Norah Salem Alsaiari, Fatimah Mohammed Alzahrani, Abdelfattah Amari, Haitham Osman, Hamed N. Harharah, Noureddine Elboughdiri, Mohamed A. Tahoon

**Affiliations:** 1 Department of Chemistry, College of Science, Princess Nourah bint Abdulrahman University, P.O. Box 84428, Riyadh 11671, Saudi Arabia; 2 Department of Chemical Engineering, College of Engineering, King Khalid University, P.O. Box 9004, Abha 61411, Saudi Arabia; 3 Research Laboratory of Processes, Energetics, Environment and Electrical Systems, National School of Engineers of Gabes, Gabes University, Gabes 6029, Tunisia; 4 Chemical Engineering Department, College of Engineering, University of Ha’il, P.O. Box 2440, Ha’il 81441, Saudi Arabia; 5 Chemical Engineering Department, Modelling Analysis and Control of Systems, National School of Engineering Gabes, University of Gabes, Gabes 6029, Tunisia; 6 Department of Chemistry, College of Science, King Khalid University, P.O. Box 9004, Abha 61413, Saudi Arabia; 7 Chemistry Department, Faculty of Science, Mansoura University, Mansoura 35516, Egypt

**Keywords:** microbial synthesis, plant-mediated synthesis, green synthesis, antimicrobial, drug delivery, anticancer, water treatment, environmental sustainability

## Abstract

The unique biological and physicochemical characteristics of biogenic (green-synthesized) nanomaterials (NMs) have attracted significant interest in different fields, with applications in the agrochemical, food, medication delivery, cosmetics, cellular imaging, and biomedical industries. To synthesize biogenic nanomaterials, green synthesis techniques use microorganisms, plant extracts, or proteins as bio-capping and bio-reducing agents and their role as bio-nanofactories for material synthesis at the nanoscale size. Green chemistry is environmentally benign, biocompatible, nontoxic, and economically effective. By taking into account the findings from recent investigations, we shed light on the most recent developments in the green synthesis of nanomaterials using different types of microbes and plants. Additionally, we cover different applications of green-synthesized nanomaterials in the food and textile industries, water treatment, and biomedical applications. Furthermore, we discuss the future perspectives of the green synthesis of nanomaterials to advance their production and applications.

## 1. Introduction

In recent years, advancements in nanotechnology have permitted the synthesis of a wide class of materials with one dimension or more in the nanoscale range (from 1 to 100 nanometers) [1,2]. These synthesized chemical materials in the nanoscale range are called nanomaterials (NMs). The difference between bulk materials and NMs is their size, i.e., bulk materials are >100 nm in all dimensions and visible through a simple microscope whereas NMs are <100 nm in at least one dimension and cannot be seen by the naked eye. In contrast to bulk materials, the physical properties of NMs are dependent on their shape and size, whereas the physical properties of bulk materials are independent of size [3]. This effect of size on the physicochemical properties (optical, mechanical, electrical, and chemical properties) of materials underlies the importance of NMs. NMs show unique properties that allow excellent performance and they have been applied in different fields, such as transportation, food safety, homeland security, information technology, environmental science, medicine, and energy [4,5,6,7,8,9,10,11,12,13,14]. The most commonly applied NMs include single/multi-walled carbon nanotubes (CNTs), titanium dioxide (TiO_2_) nanoparticles, graphene oxide nanoparticles, zinc oxide (ZnO) nanoparticles, and silver (Ag) nanoparticles. It is important to keep in mind that the majority of nanomaterials are manufactured from elements that naturally exist, such as diamond and diamondoid forms of carbon, enabling stronger products to be produced with less material.

**Figure 1:**
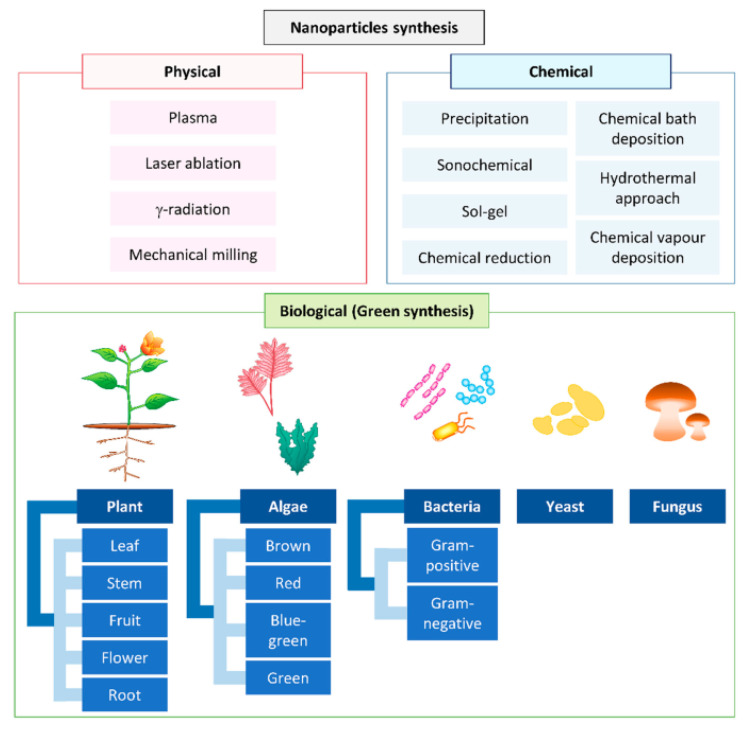
Different approaches used for the synthesis of nanoparticles. Copied from MDPI [17].

There are several methods for producing NMs that are categorized under two major approaches: the first is a bottom-up approach (chemical methods such as hydrothermal, sonochemical, electrochemical, photochemical, microwave, microemulsion, pyrolysis, redox, co-precipitation, and sol-gel methods) in which NMs are produced through the combination of single atoms or molecules [15], and the other is a top-down approach (physical methods such as lithography, ball milling, vapor and gas phase, evaporation–condensation, electro-deposition, pulsed laser ablation, arc discharge, sonication, and spray pyrolysis) in which NMs are produced by reducing large molecules to the nanoscale size [16]. Figure 1 shows the different methods used for the synthesis of nanoparticles.

**Figure 2:**
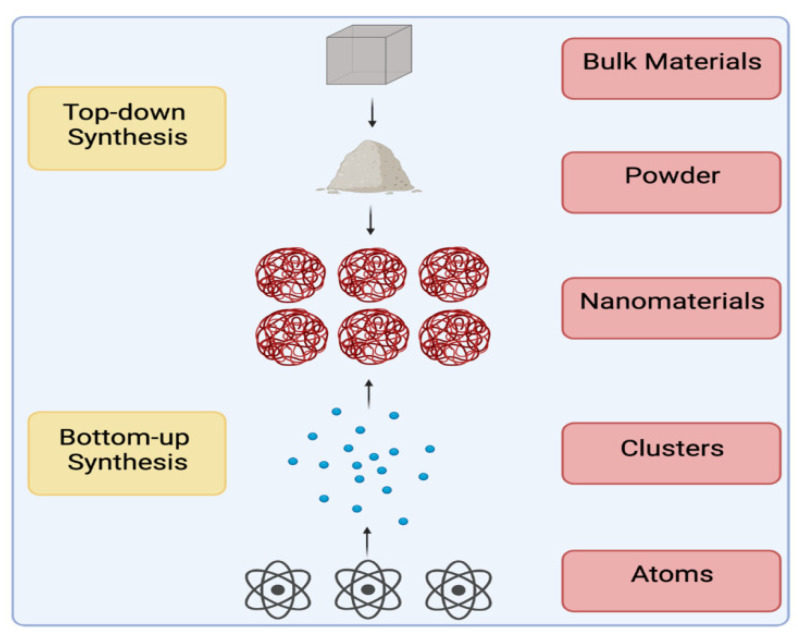
Difference between bottom-up and top-down methods used for the synthesis of NMs. Copied from MDPI [19].

Additionally, the biological method, which is a bottom-up approach, synthesizes NMs using biopolymers, biomolecules, viruses, bacteria, yeasts, plants, and fungi [18]. Figure 2 shows the difference between the two methods used for the synthesis of NMs (bottom-up and top-down). Table 1 summarizes the advantages and disadvantages of the different approaches used for the synthesis of NMs.

According to Table 1, the classical methods for the synthesis of NMs require operator skill, are expensive, and not fully understanding the modeling factors during synthesis can result in unstable NMs with environmental issues, toxic properties, and bioaccumulative behavior [20]. Subsequently, the use of NMs in different applications can cause serious problems in ecosystems, especially the marine environment, due to the release of nanoproducts in sewage sludge and industrial waste [21,22]. Over the past several years, green synthesis has grown in popularity for the research and development of NMs as a means of addressing the environmental sustainability concerns of the conventional approaches [23]. Green synthesis of NMs aims to produce NMs while reducing environmental problems associated with anthropogenic activities and inhibiting harm from known contaminants. Thus, green synthesis of NMs is eco-friendly and safe compared to classical methods since it uses biological components in the synthesis process to produce biocompatible products without using toxic chemicals. In addition, the biological components are inexpensive since they are energy intensive [24,25]. As a result, researchers have focused on the green synthesis of NMs, especially cerium oxide (CeO_2_), copper (Cu), manganese (Mn), ZnO, gold (Au), and silver nanoparticles (Ag) [26,27,28,29,30,31]. Many previously conducted reviews have focused on the classification of NMs, their synthesis, and their environmental applications, while losing focus on their toxicity, green synthesis, types of solvents used for the biosynthesis of NMs, applications of NMs in several fields, and the role of biological moieties in NM synthesis.

This paper discusses the toxicity of NMs in the environment, which reflects the different solvents utilized in NM biosynthesis. Thus, we review NM green synthesis using different biological reducing and capping agents. Moreover, we highlight the applications of NMs in different fields and briefly summarize the challenges and benefits of green-synthesized NMs, suggesting guidelines for future work. This review will support researchers working in the nanotechnology field by providing important information to guide their forthcoming research.

## 2. Nanomaterial Characterization

The properties of nanomaterials should be characterized before their use in any application. Several characterization techniques are used to determine the physical, chemical, and other properties of NMs, and these techniques depend on the type of application. By using characterization techniques, we can identify the physical and chemical properties of NMs, such as topography, roughness, surface chemistry, stability, and distribution, in addition to their surface area, shape, size, and composition. Table 2 summarizes popular characterization techniques and the obtained properties from each technique.

## 3. Toxicity and Stability of Nanomaterials

Nanomaterials are very toxic when exposed via skin penetration, ingestion, and inhalation [32]. In addition to their exposure or ingestion via different routes, their durability, size, dosage, and source are factors determining NM toxicity [33]. Moreover, NM functionalization, surface coating, crystal structure, aggregation, size, surface area, quantity, and mass are additional factors determining their toxicity. The negative health effects are increased by increased exposure to NMs via different routes, including cellular contact (drug intake and injection) and different environmental pathways. The negative health impacts include nervous system absorption and translocation to the circulatory and lymphatic systems [34,35]. Therefore, NMs should be considered as potential health dangers in nanotechnology applications. The negative impacts of NMs on the surrounding environment arise from their fine size. For example, fine nanoparticles of iron (Fe) cause apoptosis, oxidative stress, and induce the formation of reactive oxygen species due to their ability to aggregate inside organisms [36]. Additionally, the inhalation of 10 mg/m^3^ of TiO_2_ nanoparticles with a size of 20 nm caused greater lung tumor development than inhaling 250 mg/m^3^ of TiO_2_ nanoparticles with a size of 300 nm [37]. The concentration of NMs or the amount that reaches the biological system is associated with the amount in water, food, and air, which increases with increased exposure time. Si_3_N_4_, ZrO_2_, Al_2_O_3_, Fe_2_O_3_, TiO_2_, and silica are less toxic than CNTs [38]. However, the functionality, diameter, and type of CNT (single-walled or multi-walled) affect the toxicity of CNTs. In addition, zirconia, iron oxide, alumina, titania, and asbestos are less toxic than Ag nanoparticle aggregates [39]. In terms of environmental parameters, NMs are greatly affected by existing organic compounds that can cause aggregation, colloid-forming compounds, type of water (hard water or seawater), pH, and salinity.

Transformation is an important property when assessing the toxicity of NMs. For instance, the toxicity of silver nanoparticles is greatly reduced by the sulfurization process due to the ability of silver sulfide to be absorbed [20,40]. Likewise, a green approach toward engineering NMs can be achieved by introducing a biocompatible stabilizing agent. For example, outstanding stable Au nanoparticles can be obtained by coating with red ginseng root [41]. The reaction of nanoparticles with cell components can lead to a transformation process that reduces the toxicity of nanoparticles, as reported for copper oxide (CuO) nanoparticles that interacted with the cells of *Nicotiana tabacum* and underwent a transformation process with a reduction of toxicity [42]. Moreover, this work revealed that the functional groups, surface area, shape, and size of NMs greatly controlled the toxicity of NMs, especially in genetic engineering due to the modification of protein binding. This binding of NMs and protein can cause protein interruption, loss of enzymatic activity, and may produce toxic biological products [43]. Additionally, the toxicity of NMs is greatly affected by their structural features, composition, size, and shape, in addition to their surface chemistry.

Electrostatic stabilization techniques on the nanoparticle surface can be used for the stabilization process [44]. The transport and dispersion of NMs in the environment are determined by their capacity to form aqueous suspensions in different fluids. The environmental stability of nanoparticles can be assessed by their tendency to interact with close medium or their aggregation. The decrease in free energy and density of nanoparticles that result from aggregation cause a decrease in their reactivity. Subsequently, there is a serious necessity to produce safer NMs with the same unusual chemical and physical properties. Alternative novel methods have been used to synthesize novel Ag nanoparticles that are more eco-friendly than those produced using conventional methods, utilizing bacteria, plant extracts, and fungus as stabilizing agents [45]. For example, tea leaf extract was used for the green synthesis of Ag nanoparticles with good stability [46]. The size, concentration, and surface charge of Ag nanoparticles determine their toxicity. Surface coating can stabilize NMs and inhibit critical enzymes from hindering the growth of aquatic organisms. Additionally, bioaccumulation inside plant parts enables many aquatic plants to dissolve Ag nanoparticles [47]. Moreover, the size and antimicrobial features of Ag nanoparticles were investigated in the presence of carbon particles located in sulfur [48]. The stability of NMs was found to be altered by surface complexation via regulatory colloidal stability [20]. The stability and composition of NMs could be analytically predicted with insight into the surface complexation mechanisms [49]. Thus, the colloidal stability of NMs could be affected by altering their functionalization, surface coating, and size. Additional studies on NM biochemical interactions and kinetics are required to increase the knowledge of NM toxicity and stability.

## 4. Green Synthesis of Nanomaterials

Green synthesis is a subdivision of green chemistry, a science that developed as a result of the realization that chemical manufacturing needed sustainable procedures. Green chemistry aims to develop safer chemical products and procedures that reduce or eliminate the manufacture and use of hazardous components [50]. As a result, several fundamental green chemistry principles, including mitigating environmental pollution, using a renewable feedstock, using non-toxic (or safer) solvents/auxiliaries, minimizing the use of derivatives, and waste prevention or reduction, are applied in green synthesis.

**Figure 3:**
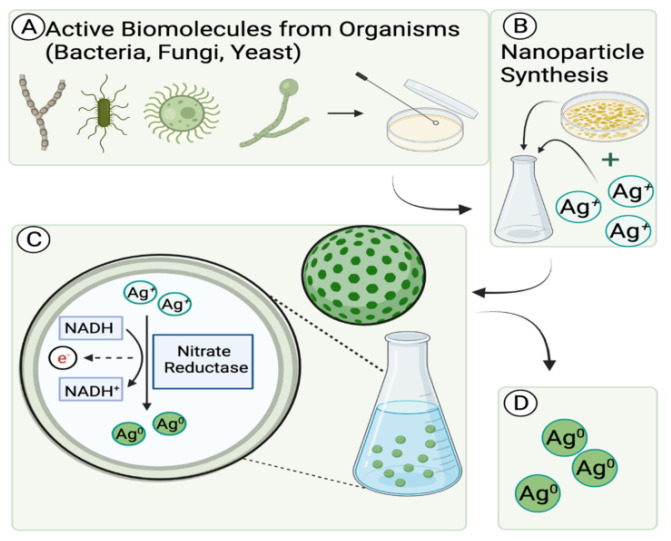
Representation of the role of active molecules in green metallic nanoparticle synthesis. (**A**) The culture of microorganisms from raw samples, (**B**) harvest of culture, (**C**) the reduction of metal ions to nanoparticles, and (**D**) collection of nanoparticles. Copied from MDPI [19].

Nanomaterials can be synthesized using plant extracts, microorganisms, and other biological contents, which is known as biological synthesis. This method is eco-friendly and cost-effective since microorganisms such as yeast, viruses, fungi, and bacteria act as nanofactories for the nano synthesis of materials [51,52]. In the presence of reductase enzymes, microorganisms can reduce metallic salts to nanomaterials [53,54]. Figure 3 shows the use of fungi, yeasts, and bacteria for the green synthesis of NMs, such as Ag nanoparticles.

Additionally, phytonanotechnology, in which NMs can be synthesized using fruits, seeds, roots, stems, and leaves, is attracting the attention of scientists [55]. Nevertheless, there are some problems associated with the biological green synthesis of NMs, such as the difficulty of recovering metallic nanoparticles, storing microorganisms, as well as their isolation, culturing, and sampling. All of these steps are complicated and hinder the green synthesis of NMs. The next subsections discuss the green synthesis of NMs using different methods.

### 4.1. Actinomycetes

A useful resource for the environmentally friendly production of nanomaterials is actinomycetes, which can use extracellular or intracellular approaches to synthesize NMs [56,57,58]. They have an intrinsic size and surface provided by a large number of secreted secondary metabolites. Several reported works synthesized NMs using actinomycetes. In recent research, *Streptomyces* spp. were used for the synthesis of white-colored and hexagonal ZnO nanoparticles with biomedical applications [59]. This research showed that only three strains from five isolated samples were effective for the synthesis of ZnO nanoparticles. These actinomycetes mediated the synthesis of ZnO nanoparticles by the reduction of particles from micro to nano size with the aid of enzymes and by metabolic reactions of the actinomycete isolates. Additionally, *Nocardia farcinica*, *Streptomyces viridogens*, *Rhodococcus* spp., *S. hygroscopicus*, and *Thermoactinomycete* spp. were used for the synthesis of Au nanoparticles [60,61,62,63]. Additionally, *Streptomyces* spp. were used for the synthesis of Mn, Zn, and Ag nanoparticles [64]. The presence of Au NPs was reduced on the cell wall and membrane as opposed to in the cytosol by the intracellular reduction of Au ions [65]. Au NP reduction was started by a mixture of enzymes released from the membrane and cell wall, and the produced particles were stabilized by proteins [65]. Additionally, the research showed that the nitrate reductase enzyme was dependent on the NADH molecule and reduced Ag ions to create stable Ag nanoparticles [66]. In recent research, CuO nanoparticles were synthesized using marine endophytic actinomycetes CKV1 as the stabilizing and reducing agent [67]. These actinomycete-mediated CuO nanoparticles had a spherical morphology with an average size of 20 nm, as revealed from TEM analysis, and exerted activity against bacteria and cancer. This research showed that actinomycetes are strong contenders for the creation of intracellular and extracellular metal and metal oxide nanoparticles because they are polydisperse and stable. Intracellular nanoparticular structures are produced when metal ions attach to the carboxylate group of an enzyme in the mycelial cell wall. The benefit of actinomycete-facilitated synthesis is that they have protein, which greatly boosts the efficiency of nanoparticle synthesis, making it simple to scale up the procedure. Another benefit is that actinomycetes are simple to extract by filtration, in contrast to other methods that demand methodical tools for careful extraction from their sources. Actinomycetes are therefore far more affordable because they do not require expensive equipment.

### 4.2. Algae

**Figure 4:**
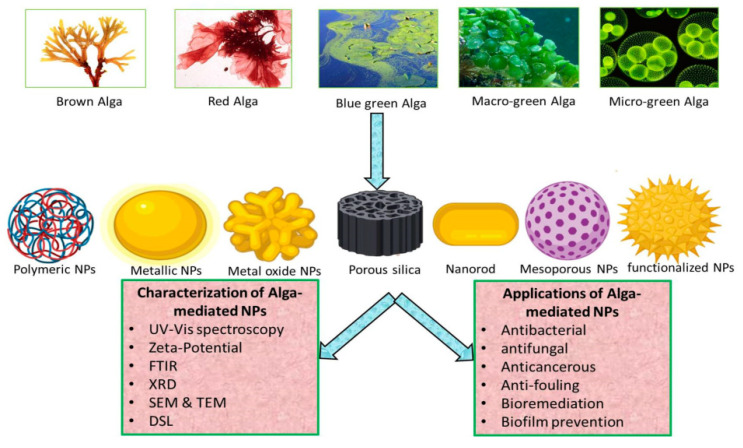
Algae-mediated synthesis of nanoparticles, their characterization, and applications. Copied from MDPI [70].

Microalgae are aquatic, photosynthetic microorganisms that can be either unicellular (such as chlorella) or multicellular (such as brown algae) [68]. Algae were discovered to have a significant role in the biological synthesis of NMs and the accumulation of different heavy metals, just like other microorganisms [69]. Figure 4 shows the algae-mediated synthesis of nanoparticles.

Algae are occasionally employed for the synthesis of zinc oxide nanoparticles and in large scale synthesis of Au and Ag nanoparticles. More often than not, microalgae are known for their capacity to convert hazardous forms of metals to their innocuous counterparts [71]. In a recent study, the microalgae strain *Phaeodactylum tricornutum* and its supernatant were used for the biosynthesis of titanium nanoparticles with a mean particle diameter of 49.7 nm [72]. These microalgae-synthesized nanoparticles had potential biomedical applications, such as imaging techniques, hyperthermia, biosensor, drug delivery systems, cancer treatments, and immune system studies because the synthesized nanoparticles had cytotoxic, antimicrobial, antistatic, and biogenic effects. Additionally, *Sargassum muticum* and *Sargassum myriocystum*, two separate species of microalgae, were used to create zinc oxide nanoparticles with a size of approximately 36 nm [73]. *S. myriocystum*-synthesized nanoparticles were found to vary in shape and size and contained carbonyl and hydroxyl groups. Further, *S. muticum*-synthesized nanoparticles were found to have polysaccharides containing hydroxyl and sulfate groups, and the nanoparticles were hexagonal. As reported in the literature, the biosynthesis of Ag NMs was assessed using two methods that incorporated the cyanobacteria and microalgae strains [74]. In one method, live biomass from the two groups of microorganisms was washed and suspended in a silver nitrate solution; in the other, the cell-free medium was added to silver nitrate. Fourteen of the sixteen strains examined were effective in producing Ag nanoparticles with sizes ranging from 13.0–31.0 nm using the two aforementioned methods, proving that the synthesis of silver nanoparticles involved the use of extracellular substances. The silver nanoparticles, except the biggest ones produced by the cyanobacterium strain (*Limnothrix sp.* 37-2-1), have also been shown to have antibacterial activity. Moreover, *Chlorella vulgaris,* as a nanofactory microalgae, was used for the synthesis of Ag and Au nanoparticles [75]. The two types of nanoparticles showed antibacterial activity toward *Staphylococcus aureus*, *Streptococcus* spp., and *Escherichia coli.* Similarly, *Chlorella vulgaris* was used for the green synthesis of tin oxide (SnO_2_) nanoparticles with photocatalytic and biological activities [76]. The synthesized nanoparticles showed photocatalytic degradation toward methyl orange dye UV radiation, antioxidant activity, cytotoxicity against lung cancer, and antibacterial activity against four pathogenic bacteria. The marine macroalgae *Padina* spp. were used to successfully demonstrate the use of marine macroalgae for the synthesis of Ag nanoparticles. It was found that the addition of this species of algae resulted in increased synthesis of Ag nanoparticles [77]. Furthermore, the nanoparticles showed strong antibacterial activity against *Staphylococcus aureus* and *Pseudomonas aeruginosa*, with inhibition zones measuring 15 and 13 mm, respectively.

### 4.3. Plant-Mediated Synthesis

**Figure 5:**
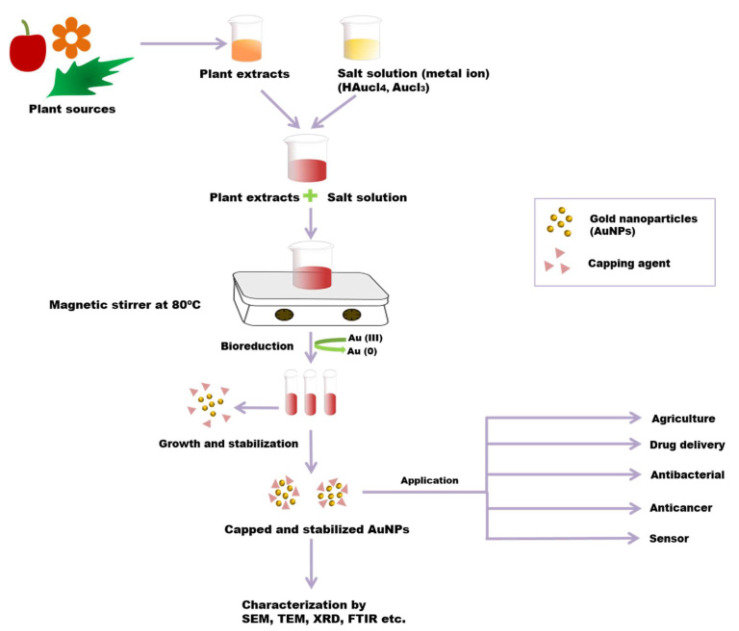
Plant-mediated synthesis of Au nanoparticles and their characterization. Copied from MDPI [79].

Compared to other green synthesis methods of NMs, plant-mediated synthesis of NMs is the most efficient due to the high resultant yield. The high yield of NMs results from the high stability of synthesized NMs in different species of plants. The different metabolites and biochemical species of plants are responsible for the stabilization of NMs [78]. Thus, plant-mediated synthesis of NMs is a cost-effective (economical) as well as eco-friendly method. Figure 5 shows a schematic diagram for the use of plant extracts to synthesize nanoparticles, such as Au nanoparticles, and their characterization.

Plant-mediated synthesis of NMs can be classified as intracellular, extracellular, and phytochemical synthesis. The extracellular method is the most effective and produces a higher yield of NMs than the intracellular method. This is may be attributed to the high number of phytochemicals in the plant extract, which play an essential role in the synthesis process. Meanwhile, the essential role of synthesis in the intracellular method is attributed to the cellular enzymes of different plant tissues, which later require complicated steps to attract the synthesized NMs by cracking the cell wall of the plant. Recently, aqueous leaf extracts of *Lantana camara* were used for the green synthesis of yttrium oxide (Y_2_O_3_) nanoparticles ranging from 20 to 45 nm [80]. The synthesized Y_2_O_3_ nanoparticles showed photocatalytic activity toward the degradation of Rhodamine B (RhB) dye under a 250 W Xenon lamp with high pressure. Additionally, the synthesized Y_2_O_3_ nanoparticles showed antibacterial activity toward Gram-negative bacteria, such as E. coli (MTCC 732), and Gram-positive bacteria, such as *Bacillus subtilis* (MTCC 5981). Moreover, these nanoparticles showed cytotoxic effects against HeLa cell lines and can be used to treat human cervical cancer. The results showed that these Y_2_O_3_ nanoparticles had strong surface reactivity, making them good candidates in cancer therapy to deliver targeted drugs. Therefore, these green-synthesized nanoparticles had appropriate features for use in different medical applications. In other research, aqueous extracts of the root, stem, and leaf of *Capsicum chinense* were used for the synthesis of Ag and Au nanoparticles, with the best results obtained with the leaf extract [81]. The leaf-derived Ag nanoparticles were synthesized using UV light radiation, while the leaf-derived Au nanoparticles were synthesized using microwave radiation. The authors found that the leaf extract successfully generated stable Ag and Au nanoparticles. The results showed that the sugar content in the leaf extract was reduced by 68% after the synthesis of Au nanoparticles, while the amino acid, sugar, and polyphenol levels were reduced by 47%, 39%, and 15%, respectively, after the synthesis of Ag nanoparticles. These findings implied that these chemical groups were involved in the stabilization of the nanoparticles. The growth of *E. faecalis*, *S. marcescens*, *E. coli*, and *S. aureus* was inhibited using the Ag nanoparticles synthesized using the *Capsicum chinense* leaf extract. The results indicated that the Ag nanoparticles synthesized using *Capsicum chinense* leaf extract were good candidates for applications in the medical field. A leaf extract of *Diospyros lotus* was successfully used for the rapid and green synthesis of Ag nanoparticles [82]. This work explored the stabilization and reduction of nanoparticles by phytochemicals in plant extracts, such as terpenoids, tannins, and steroids, and this role was confirmed by phytochemical screening and FT-IR analysis. The plant-synthesized nanoparticles had an average diameter of 20 nm, with a spherical shape demonstrated by electron microscopy. Additionally, these synthesized nanoparticles were found to have catalytic activity in reducing methylene blue dye and antibacterial activity toward *Escherichia coli*. Intriguingly, this investigation revealed that Ag nanoparticles greatly weakened platelet coagulating ability and actively reduced their activity. The leaf extract of *A. graecorum* was used for the green synthesis of Ag nanoparticles and their antitumor and antifungal activities were evaluated [83]. The results of SEM analysis showed that the size of nanoparticles ranged from 22 to 36 nm with a spherical shape. The synthesized nanoparticles showed strong antifungal activity against *C. krusei*, *C. tropicales*, *C. parapsilosis*, *C. glabrata*, and *C. albicans*. Additionally, *A. graecorum* Ag nanoparticles inhibited the growth of the MCF-7 breast cancer cell line. Thus, these green-synthesized nanoparticles had potential as antifungal and antitumor agents for several therapeutical applications. Recently, Ag nanoparticles with an average particle size of 26.63 nm were synthesized using the leaf extract of *Origanum majorana,* demonstrating antibacterial efficiency against multidrug-resistant bacterial strains [84]. The results showed that these Ag nanoparticles were highly efficient antimicrobial coatings on environmental surfaces in hospitals and intensive care units, thus inhibiting the extent of multidrug-resistant nosocomial bacterial contamination. In other work, *Azadirachta indica* aqueous leaf extract was used for the synthesis of Ag nanoparticles via an eco-friendly, non-toxic, quick, and simple one-step method [85]. According to this method, the creation of silver nanoparticles from silver ions took 15 min at room temperature. Given that no potentially harmful chemicals were used, this method was eco-friendly. When tested against the Gram-negative and Gram-positive bacteria *Staphylococcus aureus* and *Escherichia coli*, the synthesized Ag nanoparticles demonstrated an antibacterial effect. Plant-synthesized Ag nanoparticles are very popular for the treatment of cancer and viral infections. The unique properties of nanoparticles, such as their shape, size, and surface charge, are crucial because these directly affect how well they perform biological tasks. Studying the pharmacodynamics and pharmacokinetics is important to understand how these nanoproducts and their properties help treat diseases like cancer. Additionally, prolonged in vivo investigations of nanoparticles should be carried out [86].

### 4.4. Viruses

Over the last decade, virus-mediated biosynthesis of NMs has been used in different applications [87,88]. The virus’s outer capsid protein serves an appealing purpose in the production of NMs by providing a very reactive surface that interacts with metal ions [89]. There are 2130 capsid proteins on the tobacco mosaic virus (TMV) that cover its surface and serve as notch additions for the material to bond or can be used to introduce 3-D vessels for use in a variety of medicinal applications [90,91]. Before incorporating plant extracts of *Hordeum vulgare* (barley) or *Nicotiana benthamiana* (round-leaved native tobacco), low quantities of TMV resulted in a decrease in the size of the produced nanoparticles when Ag or Au salts were used. Additionally, TMV increased their amounts in comparison to those without viral addition; on the other hand, less free nanomaterial was produced when there were more viruses present in the reaction environment [92]. Recently, Au and Ag hybrid NMs were synthesized using a nanoscale biotemplate (32 nm plant virus) of squash leaf curl China virus [93]. These eco-friendly hybrid NMs were synthesized after only 5.0 min of mixing gold chloride and silver nitrate solutions with the virus in sunlight. The metallic hybrid (Ag and Au) NMs were verified to exhibit excellent electrical conductivity inside the semi-conductive band gap, producing effective bio-semiconductors for biomedical applications. The metallization of nanowires was also achieved using TMV as a biotemplate. It has been stated elsewhere that viruses have untapped potential for producing several types of nanostructures [94,95]. They produce important inorganic materials, such as iron oxide (Fe_2_O_3_), zinc sulfide (ZnS), silicon dioxide (SiO_2_), and cadmium sulfide (CdS). Some of these materials are very important in the electronics industry as they are used in electronic products.

### 4.5. Fungi

**Figure 6:**
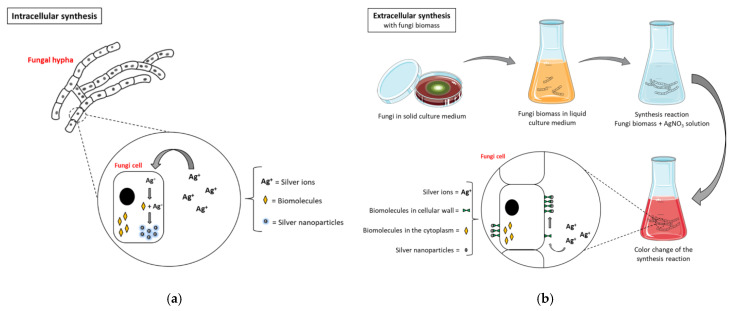

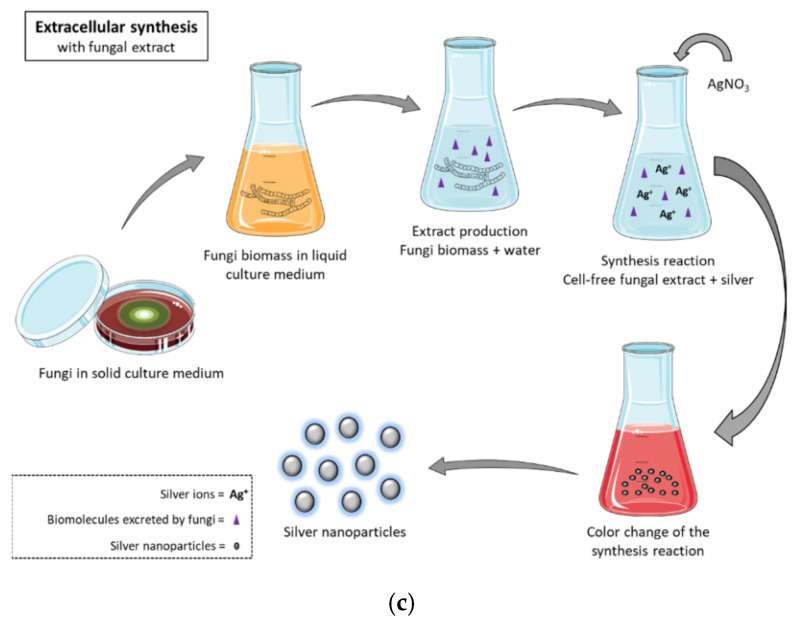
Intracellular biosynthesis of Ag nanoparticles using fungi (**a**), extracellular biosynthesis of Ag nanoparticles using fungal biomass (**b**), and extracellular biosynthesis of Ag nanoparticles using cell-free fungal extract (**c**). Copied from MDPI [99].

Many research teams throughout the world have used fungus to biosynthesize nanoparticles, and the process takes place both extracellularly and intracellularly. For instance, the ability of fungi such as *Penicillium* spp., *Fusarium* spp., *F. oxysporum*, *F. semitectum*, *F. acuminatum*, *F. solani*, *Cladosporium cladosporioides*, *T. asperellum,* and *Aspergillus* spp. to synthesize both Au and Ag nanoparticles has been regularly reported [96,97,98]. Figure 6 shows the differences between intracellular and extracellular biosynthesis of nanoparticles, such as Ag nanoparticles, using fungi.

Additionally, research has demonstrated that fungi can produce mono-dispersed NMs in a variety of chemical compositions and particle sizes. When compared to other microorganisms, such as bacteria, fungi have a few extra qualities that help with the production of metallic NMs. For example, fungi secrete enormous quantities of enzymes and proteins per unit of their mass, leading to the production of a higher yield of NMs than bacteria. According to many studies, some fungi produce smaller sized, synthesized particles due to their high intracellular metal absorption volumes [100]. However, the culture conditions might have a substantial impact during the biosynthesis of metallic NMs. For instance, *Trichothecium* spp. biomass was used to create extracellular NMs during the biological reduction of gold ions in stationary environments. The biomass, on the other hand, tended to form intracellular NMs when it was stirred up. This discovery raised the possibility that non-stirring conditions encouraged the release of proteins and enzymes, whereas stirring inhibited their liberation [101]. According to fluorescence spectra investigations, bioactive reducing chemicals secreted from the cell wall acted as catalysts for the extracellular synthesis of nanoparticles by fungi, which resulted in the production of protein-stabilized NMs. The research demonstrated that the solution contained identical proteins generated by the fungus biomass and also bonded to the NM surfaces [102]. Researchers have investigated the intracellular and extracellular production of NMs utilizing fungus biomasses. A low quantity of produced NMs is a disadvantage of the extraction processes used in downstream processing for intracellular production. Conversely, extracellular production results in NMs near the cell surface or at the cell’s periphery, which makes them easy to retrieve during subsequent processing [103]. The capacity of some fungi to generate NMs with various chemical structures is particularly noteworthy. For instance, aqueous solutions of TiF_6_^2−^ and Si_6_^2−^ were used to biosynthesize titania and silica nanoparticles, respectively, using *Fusarium oxysporum* [104]. Additionally, the previous studies confirmed the ability of fungi to produce a variety NMs, such as metal oxide NMs, metals, magnetic nanoparticles, and quantum dots [105,106,107,108].

### 4.6. Yeast

Yeasts are microorganisms belonging to single-cell eukaryotes that have evolved from multicellular antecedents, and various studies have shown that yeasts can be used to successfully synthesize NMs. There are approximately 1500 species of yeast, many of which are extensively used in the production of metallic NMs. Due to their wide surfaces, yeasts can absorb and accumulate a significant amount of dangerous metals from their surroundings [109]. Yeasts adapt to hazardous metals through a variety of detoxifying methods, including biosorption, extracellular sequestration, chelation, and bioprecipitation. These methods, which yeast cells have developed, are employed to create NMs and make them more durable, leading to differences in particle characteristics and size. Several studies have been conducted to investigate the synthesis of metallic NMs using yeast. Recently, M. Rasouli showed that the yeast *Nematospora coryli* can uptake the oxyanions of Se and intracellularly convert them to zero-valent Se (Se^0^) nanoparticles with a spherical shape and size of 50–250 nm [110]. The results confirmed the biological activities of the yeast-synthesized Se^0^ nanoparticles. Further, this method was cost-effective and did not use any hazardous materials. Similarly, baker’s yeast (*Saccharomyces cerevisiae*) was used to convert sodium selenite intracellularly into amorphous-shaped Se nanoparticles that were 71 nm in size [111]. These yeast-derived Se nanoparticles were bio-inert, gray, or black instead of red Se nanoparticles, and they could be safely used to increase animal defense against infectious diseases and oxidative stress as a trace element feed additive. Additionally, *S. pombe* was used for the intracellular synthesis of semiconductor CdS nanoparticles with high forward current value and low-voltage operation, which could enhance their use in diodes [112]. Moreover, yeast strains have been used for the synthesis of Au and Ag nanoparticles. The hexagonal Ag nanoparticles were synthesized extracellularly using silver tolerant yeast strain *MKY3* with a size of 2–5 nm [113]. Standardization and recording of the ideal conditions for the synthesis of high-quantity silver nanoparticles were also carried out under various thermal conditions [113]. Additionally, chloroauric acid concentrations were varied throughout the incubation of *Yarrowia lipolytica* cells, and cell-related gold nanoparticles and nanoplates were produced [114]. This study discovered that the number of cells and salt concentrations can impact nanoparticle size [114]. Yeast was used as a biotemplate to synthesize mesoporous zirconium phosphate with excellent electrocatalytic activity for oxygen reduction reaction, allowing its use in the fabrication of air electrodes [115]. Moreover, PbS quantum dots were intracellularly synthesized using Pb^2+^ ions when mixed with the yeast cells of *Torulopsis* spp. [116]. Subsequently, the use of yeast as a biotemplate for the synthesis of different nanoparticles has become a vital, cost-effective, and eco-friendly method with various potential applications.

### 4.7. Bacteria

Many new NMs can be produced using various species of bacteria. The two main strategies for employing bacteria in the production of NMs are extracellular and intracellular. The extracellular method of NM synthesis is recognized to have advantages over the intracellular method since it does not require complicated steps to extract NMs from the mediated bacteria, in addition to the shorter time of synthesis [117]. The choice of bacterial species depends on several factors, such as the cost, eco-friendly production, and large-scale synthesis of NMs. The reported literature confirms the ability to produce metallic nanoparticles via cultures of *Actinobacter* spp., *Escherichia coli*, *Klebsiella pneumonia*, *Lactobacillus* spp., *Bacillus cereus*, *Corynebacterium* spp., *Pseudomonas* spp., and *Enterobacter cloacae*, which reduce the metallic ions to nanoparticles [118,119,120,121]. Nevertheless, there were limited shapes and sizes of the bacteria-synthesized nanoparticles compared to those produced using other conventional methods. In this section, bacteria-mediated synthesis of nanoparticles is discussed. *Morganella morganii* was utilized for the synthesis of Cu nanoparticles, which was achieved by the reduction of Cu ions absorbed intracellularly into metallic (Cu^0^) by means of a certain protein or metallic ion reductase [122]. This is a perfect way to synthesize Cu^0^ nanoparticles as they are reportedly unstable and rapidly oxidized to the oxide form (CuO) [122]. As copper metallic nanoparticles flow out of the cell, they accumulate outside the cell. Silver nanoparticles were also synthesized extracellularly using *Morganella* spp. [123]. Additionally, *B. licheniformis* was used for the synthesis of ZnO nanoflowers with photocatalytic properties toward the degradation of dyes such as methylene blue. When compared to other photocatalytic substances, these nanoflowers demonstrated higher photocatalytic activity, and it has been hypothesized that this is due to the greater oxygen vacancy present in the produced nanoparticles. These synthesized nanoflowers were 400 nm in height and 40 nm in width [124]. *Rhodococcus* can aid in biodegradation since it can persist in harsh conditions and can metabolize hydrophobic substances [125]. Thus, *Rhodococcus pyridinivorans* was used to synthesize spherical ZnO nanoparticles with a size of 100–130 nm using zinc sulfate as a substrate [126]. *A. hydrophilla* was used for the synthesis of ZnO nanoparticles with a size of 42–64 nm and different shapes, such as spherical and oval [127]. According to reported studies, *Pseudomonas aeruginosa rhamnolipid*-stabilized ZnO nanoparticles exceeded bare ZnO nanoparticles in terms of antioxidant activity because it is difficult to form micelle aggregates on carboxymethyl cellulose [128]. Additionally, due to its lengthy carbon chain, it performed better as a capping agent [129]. This work demonstrated the production of ZnO nanoparticles with a size of 27–81 nm and spherical shape [129].

As one of the platinum group metals (PGM), palladium (Pd) is used as a catalyst for hydrogenation and dehalogenation reactions because it can combine with highly catalytically active metals. Zero-valent palladium (Pd^0^) nanoparticles were produced as a result of heavy metal contamination of bacteria isolated from Alpine sites [130]. Among the numerous bacteria isolated from the site, only *Pseudomonas* cells showed the ability to create catalytically active Pd nanoparticles. In addition, they were able to dehalogenate tri- and tetra-chlorinated dioxin using reductive means. Hydrogenases found within the cells of *Escherichia coli* were used to synthesize Pd^0^ nanoparticles [131]. Pd nanoparticles were created on the bacterial cell membrane and were simple to separate.

Because of their biological activities, studies have focused on the eco-friendly synthesis of silver and gold NPs. Lactic acid bacteria were used for the green synthesis of silver nanoparticles [132]. *Lactococcus garvieae*, *Enterococcus faecium*, *Pediococcus pentosaceus*, and *Lactobacillus* spp. were used to synthesize nanoparticles. A two-step process was presented for the silver nanoparticle production process. Silver nanoparticles were formed as a result of the reduction of metallic ions after their biosorption on the cell wall [132]. The cell wall may also act as a capping agent, preserving their stability by preventing aggregation of the synthesized nanoparticles. *Bacillus licheniform* was used for the intracellular synthesis of Ag nanoparticles [133]. The transformation of the culture’s color to dark brown with the addition of Ag ions served as a demonstration that the development or synthesis of nanoparticles required 24 h. However, an additional extraction step was necessary because the nanoparticles were created intracellularly. Similarly, *Bacillus* spp. cultured in silver nitrate-containing media was used for the intracellular synthesis of silver nanoparticles within 7 days [134]. Due to the straightforward purifying method and higher production rate, extracellular generation of nanoparticles is advised over intracellular synthesis [135]. In recent research, Au nanoparticles were synthesized extracellularly using the marine bacterium *Paracoccus haeundaensis BC74171^T^* with a size of 21 nm and spherical shape [136]. The existence of Au nanoparticles was confirmed by UV-VIS analysis with the appearance of an absorbance peak at 535 nm and by visual observation when a ruby red color appeared. The interaction of functional groups was determined and the presence of biomaterial on the gold nanoparticle surface was confirmed by FT-IR analysis. Cell-free supernatant-mediated Au nanoparticles showed antioxidant activity and exerted an antiproliferative effect against normal and cancer cells. The biosynthesis of Au nanoparticles was achieved using *Micrococcus yunnanensis J2,* which was isolated from mine soil samples and identified using 16S rDNA sequencing [137]. The results showed that the biogenic Au nanoparticles were spherically shaped with a size of 54 nm. The cytotoxic activity of the biogenic Au NPs was assessed, and the results showed that normal cells were less affected than cancer cell lines. However, compared to the biogenic Au NPs, Au^3+^ ions showed more toxicity against both normal and cancer cells. Inspection of antibacterial activity revealed that Au^3+^ ions had a greater inhibitory effect than Au NPs. *Enterococcus* spp. was used for the intracellular synthesis of Au nanoparticles with a spherical shape [138]. According to this study, the specific ions were transported into the negatively charged cell wall and diffused through the cell wall by electrostatic attraction with the positively charged metals. Subsequently, the toxic metals were converted to non-toxic metallic nanoparticles by the action of presented enzymes in the cell walls of the microbes. *Enterococcus* spp.–mediated Au nanoparticle induced cytotoxicity in human colorectal cancer cells and could be used as a pro-apoptotic agent for colon cancer treatment. Moreover, the non-involvement of biological enzymes in the synthesis of nanoparticles was also reported in many studies. For instance, nanoparticles were synthesized using *Corynebacterium* spp. via a non-enzymatic reduction mechanism [139]. Similarly, dried cells of *Bacillus megaterium* produced Au nanoparticles [140]. This reduction process was explained by the effect of environmental parameters, such as temperature and pH, and by the existence of some organic functional groups on the cell wall that induced reduction [141]. For example, functional groups present on the cell wall of *Bacillus megaterium D01* and *Lactobacillus* spp*. A09* interacted with Ag ions to produce Ag nanoparticles [142]. In the overall assessment of bacteria-mediated synthesis of nanoparticles, bacteria have extensive potential for the synthesis of nanoparticles because of their quick replication rate and relatively simple cultivation technique. The synthesized nanoparticles using bacteria are eco-friendly and may have several medicinal applications. Table 3 summarizes the microbial synthesis of NMs using different types of organisms.

## 5. Different Applications of Nanomaterials

**Figure 7:**
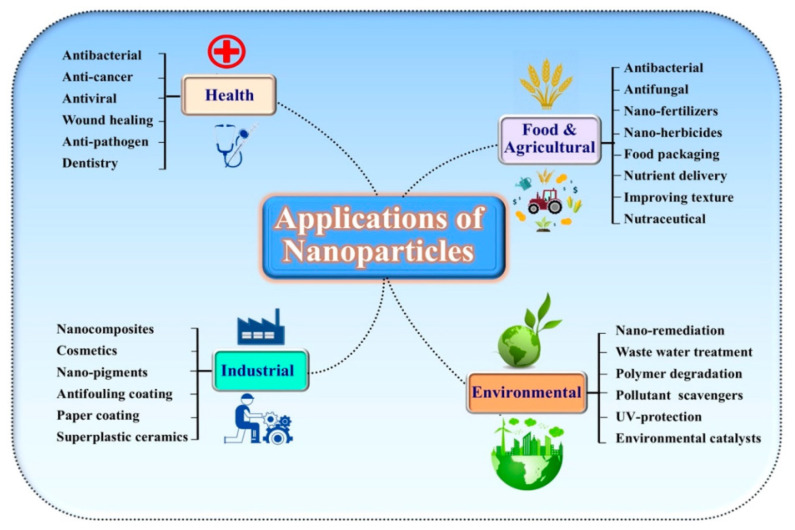
Use of nanoparticles in different fields. Copied from MDPI [143].

Green-synthesized NMs have numerous uses in physicochemical and biological applications. In biomedical research, they could be applied for biomolecular recognition, bioimaging, biosensors, and drug delivery. Because of their biological activities, these green-synthesized NMs can be incorporated into different everyday materials, such as humidifiers, water purification systems, deodorant, toothpaste, and cosmetics. Additionally, they can be used for energy storage in oxide and solar batteries. Further, NMs have a significant impact in the agricultural field, helping to identify and eradicate plant infections and reduce nutrient loss to boost crop productivity. Moreover, the use of biodegradable materials in place of plastics and the reduction of harmful chemicals in manufacturing processes are only a few of the innovative methods offered by nanotechnology to reduce pollution in various operations. Further, its role in the treatment of water polluted with different types of contaminants, such as heavy metals, dyes, and drugs, can help preserve clean water supplies worldwide. Subsequently, nanotechnology is a hot topic now and will be in the future, and researchers and governments have high hopes for this technology’s ability to solve today’s challenges. Figure 7 shows the applications of nanoparticles in different fields.

In the next sections, we discuss the applications of NMs in different fields to provide a reference for future work.

### 5.1. Food Industry

The food industry has made extensive use of nanoparticles. Nanoparticles are heavily used in packaging, food additives, and sensors from manufacturing to nutrient delivery. However, little is known about their potential toxicity and how biomolecules and food-grade nanoparticles interact. The European Union and the United States both have a food additive registration for amorphous silica (SiO_2_). It mostly acts as an anti-caking agent in powdered and granulated products [144]. These particles help products flow well and prevents the formation of lumps. SiO_2_ may make up 2% of the total weight of food, according to the US FDA [145]. Nano-SiO_2_ was blended with low-density polyethylene to produce a container of nano-SiO_2_-modified low-density polyethylene that was used to store Pacific white shrimp (*Penaeus vannamei*) for 8 days at 4 °C with improved results compared to classical packaging requirements [146]. These improved results were attributed to the reduction of nitrogen content and thiobarbituric acid-reactive substances and the inhibition of polyphenol oxidase activity. Thus, the use of SiO_2_ nanoparticles improved the quality of food packaging. Zinc is an important trace element for humans, so zinc oxide (ZnO) nanoparticles can provide this crucial trace mineral in food supplements [147]. Because ZnO NPs have antibacterial qualities, they are also utilized in food packaging. This aids in preventing bacterial food contamination [148]. ZnO nanoparticles can also be used to safeguard foods that are sensitive to UV radiation by absorbing UV radiation [149]. Additionally, Ag nanoparticles can be used in the food industry in a variety of ways. They are frequently used during packaging and in food as antibacterial agents [150] to improve the safety and shelf life of food. Silver nanoparticles have been incorporated into plastic food boxes developed by several companies, including Sharper Image (Fresher Longer, USA), Blue Moon Goods (Fisher Scientific, USA), and A-DO Global (South Korea). Silver zeolites have been used in commercial active packaging systems by Agion Technologies, which have been approved for use by the European Food Safety Authority [151]. Additionally, the food industry employs magnetic nanoparticles (Fe_2_O_3_) as coloring agents. Titanium dioxide nanoparticles (TiO_2_) are well-known nanoparticles used as a food ingredient and approved by the US Food and Drug Administration. Nevertheless, it cannot exceed 1% of the food’s overall bulk. Moreover, the photocatalytic activity of TiO_2_ nanoparticles allows their use in the packaging of foods, exerting strong antibacterial activity. Titanium dioxide (TiO_2_)-coated packaging films are effective at preventing the contamination of food contact surfaces [152]. The highest quantities of TiO_2_ nanoparticles are found in several food products, such as gums, candies, and sweets [153]. The beneficial properties of TiO_2_ nanoparticles, such as slippery mouthfeel, high refractive index, strong coverage, and good whiteness compared to bulk TiO_2_ particles makes the nano form of TiO_2_ more suitable for improving food quality.

### 5.2. Water Treatment

Currently, the world suffers from pollution of water sources by dangerous pollutants, such as heavy metals, dyes, and pharmaceutical products [154,155,156,157]. The pollution of water sources by such pollutants results from the release of contaminated wastewater directly into these water sources. The treatment of wastewater before its release is the only way to solve this problem. The limitations of classical methods, such as incomplete removal of pollutants and long processing time, hinder their use for water treatment. Remediation of contaminated water can be efficiently achieved using green-synthesized NMs. Several types of NMs have been applied for water treatment. Eco-benign and cost-effective approaches have been used for the green synthesis of magnetic iron oxide (Fe_3_O_4_) nanoparticles applied to the photocatalytic degradation of organic dyes [158]. The seed extract of pomegranate (*Punica granatum*) has been used for the synthesis semi-spherical and uniformly distributed nanoparticles within the size range of 25–55 nm. Under UV light irradiation, the produced Fe_2_O_3_ nanoparticles had outstanding photocatalytic activity against reactive blue dye, and a maximum degradation of 95.08% was attained in 56 min of reaction time. Similarly, Fe_3_O_4_ nanoparticles have been synthesized using papaya (*Carica papaya*) leaf extract [159]. The synthesized nanoparticles were environmentally friendly and investigated for the photocatalytic degradation of remazol yellow (RR) dye with a removal capacity of 340 mg/g. Therefore, the green-synthesized Fe_3_O_4_ nanoparticles could be used for dye degradation in wastewater. CuO and ZnO nanoparticles with sizes of 30–40 nm and 20–40 nm, respectively, were synthesized using *Elaeagnus indica* leaf extract as the reducing and stabilizing agents [160]. These green-synthesized nanoparticles showed excellent photodegradation efficiency toward methylene blue dye.

**Figure 8:**
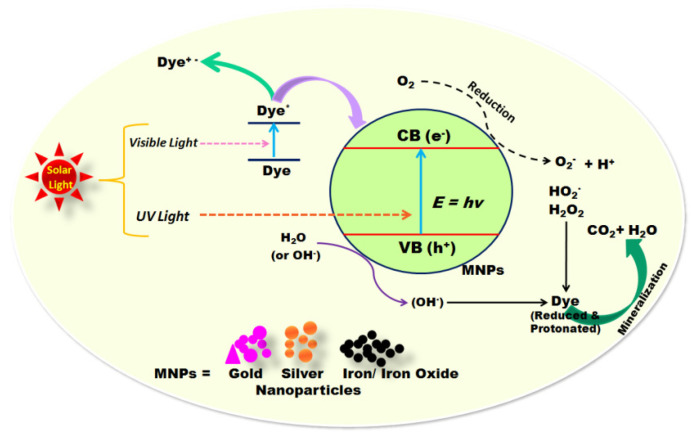
Possible mechanism for the photocatalytic degradation of different dyes using nanoparticles. Copied from MDPI [163].

In a recent study, *Camellia sinensis* (tea leaves) extract as a reducing agent was used for the green synthesis of magnesium oxide (MgO) nanoparticles with a size of 35 nm [161]. The green-synthesized nanoparticles exhibited over 97% photocatalytic degradation efficiency of methylene blue (MB) dye. In addition, green-synthesized MgO nanoparticles with an average size of 68.06 nm using the marine brown algae *Sargassum wighitii* as the reducing and capping agents showed photocatalytic activity under sunlight and UV irradiation toward methylene blue dye (MB) [162]. Overall, the results indicated that the MgO nanoparticles generated using biogenic sources exhibited potential for the degradation of organic dyes in wastewater. Figure 8 shows the general mechanism for the photocatalytic degradation of different dyes using various nanoparticles, such as Fe_3_O_4_, Fe, Ag, and Au nanoparticles.

Moreover, green-synthesized nanoparticles have been applied for the removal of toxic metals. The eco-friendly, economical, simple, and non-toxic method was used for the synthesis of TiO_2_ nanoparticles with a spherical shape and size of 10 nm using the leaf extract of *Syzygium cumini* as a capping agent [164]. These nanoparticles were examined for photocatalytic lead removal from explosive wastewater (83%) and reduction of chemical oxygen demand (COD) (75.5%). In similar research, the leaf extract of *Jatropha curcas L*. was used to produce TiO_2_ nanoparticles utilizing the polyphenolic tannins in the leaf extract as a capping agent [165]. In this work, the TiO_2_ nanoparticles reduced COD by 82% and removed chromium (Cr) ions by 76% using a self-designed solar-photocatalytic parabolic trough reactor. The results demonstrated that the green-synthesized TiO_2_ nanoparticles has remarkable photocatalytic activity and could be applied for water treatment. Therefore, we may conclude that nanoparticles have amazing potential for use as an alternative and environmentally beneficial wastewater treatment method.

### 5.3. Textile Industry

Unique chemical, physical, electronic, mechanical, and electrical properties have been observed for NMs. Due to their exceptional properties, they have become commonly used for finishing and coating textile fabrics. Microbes can destroy textile fabrics. Thus, antimicrobial textile fabrics have been produced using NMs to avoid the destruction of these fabrics. However, the toxicity of nanoparticles can hinder their application in the textile industry. Because of this, environmentally friendly nanoparticles are currently employed to create fabrics with antimicrobial characteristics. Silver nanoparticles are known to have biological activities that enhance their application in the production of medical textiles with new properties, such as bacterial pathogen self-cleaning. Recently, the roots of the medicinal plant *Achillea fragrantissima* were used to isolate the endophytic actinomycetes strain *Streptomyces laurentii R-1* for the green synthesis of silver nanoparticles used in the textile industry [166]. The synthesized nanoparticles had a spherical shape and the loading safe dose on cotton fabric was 100 ppm. The nano-finished fabric showed broad-spectrum activity toward pathogenic bacteria, even after 10 washing cycles. Moreover, silver nanoparticles have shown antibacterial properties against *E. coli,* allowing their wide application in textile fabrics [167]. Additionally, gold nanoparticles have been applied in the textile industry to improve fabric properties such as antistatic, antimicrobial, resistance to wrinkles, UV protection, flame-retarding ability, hydrophobicity, and self-cleaning properties. The extract of *Acorus calamus rhizome* was used for the green synthesis of spherical, nano-sized gold nanoparticles using chloroauric acid as a precursor to be coated on cotton fabric using the padedryecure method [168]. The results showed the antibacterial activity of gold-coated cotton fabric against both Gram-positive bacteria (*S. aureus*) and Gram-negative bacteria (*E. coli*) in addition to enhanced UV-blocking properties. The gold-coated cotton fabric gave improved Raman signals of dyes on the fabric, UV-blocking ability, and antibacterial activity. Green-synthesized gold nanoparticles are biocompatible by nature, adding to the comfort and safety of human-wearable fabrics. Zinc oxide nanoparticles (ZnO) are another metal oxide nanoparticle used in the textile industry, which produces resistance to microbial infection and high UV protection when coated on cotton fabric. Biogenic zinc oxide nanoparticles have been synthesized using *Actinobacteria Rhodococcus pyridinivorans NT2* with a size of 100–120 nm and have been coated on cotton fabric [128]. The results showed that ZnO-coated cotton fabric had improved antibacterial properties against Gram-positive *S. aureus* and epidermidis NCIM 2493 (ATCC 12228), UV-blocking ability, and a self-cleaning property against malachite green dye. Similarly, green-synthesized ZnO nanoparticles using *Acalypha indica* leaf extract as the reducing agent with a size of 20 nm were coated on cotton fabric, displaying an enhanced resistivity to microbial infection against *S. aureus* and *E. coli* and UV protection [169]. In addition, isolated *Aspergillus terreus* fungus was used as a capping agent to produce ZnO nanoparticles with a size of 10–45 nm that were used to coat cotton fabric [170]. The ZnO-coated cotton fabric showed an increase in UV-blocking ability and antibacterial activity against Gram-positive and Gram-negative bacteria with a safe dosage of 20 ppm. Copper oxide nanoparticles were also applied in different applications in recent years in the textile industry. However, copper oxide nanoparticles synthesized by chemical and physical methods are toxic. Hence, green methods can overcome this problem by producing eco-friendly, stable, and biocompatible copper oxide nanoparticles. *Ruellia tuberosa* (Snapdragon root or Minnie root) was used for the green synthesis of copper oxide nanoparticles with a size of 83 nm [171]. The copper oxide nanoparticles showed an antimicrobial effect against clinical pathogens (*S. aureus*, *E. coli*, and *K. pneumoniae*) when coated over cotton fabric. Furthermore, green-synthesized nanoparticles have been applied in the textile industry to degrade organic dyes embedded in the colorization process since these dyes are toxic to humans and the environment. These dyes can damage the liver and kidneys of animals and humans, disrupt the central nervous system, cause skin irritation and blood disorders, and can hinder the photosynthetic processes of aquatic organisms. Several green-synthesized nanoparticles have been examined for the degradation of textile organic dyes. *Tinospora cordifolia* (giloy), as a capping agent, was used for green-synthesized copper nanoparticles to be used in the degradation of dyes released by the textile industry, such as direct dye, safranin, reactive dye, and eosin yellowish [172]. *Camellia sinensis* was used for the green synthesis of SnO_2_ nanoparticles with sizes of 4.7, 5.2, and 6.91 nm and a quasi-spherical shape [173]. The synthesized SnO_2_ nanoparticles had photocatalytic activity toward the degradation of textile dyes Rhodamine B, Methyl Orange, and Methylene Blue, with percentages of 100%, 81%, and 100%, respectively. However, the use of nanoparticles to degrade textile dyes is an expensive and time-consuming process since it requires the use of a photoreactor [174].

### 5.4. Mutagenicity, Autophagy, and Cytotoxicity

Green-synthesized nanoparticles have been applied as anti-mutagenic compounds. DNA may be harmed by mutagenic materials that chemically interact with it. Humans regularly come into contact with mutagenic materials which causes great concern. A contributing cause of cancer and other disorders is DNA damage. To avoid this risk, one strategy is to create anti-mutagenic substances. Nanoparticles are widely applied as anti-mutagenic substances. *Rosa canina* waste seed was used as the reducing and stabilizing agents for the green synthesis of spherical and rod-shaped Ag nanoparticles with an average size of 150 nm [175]. These Ag nanoparticles showed good anti-mutagenic properties toward *Salmonella typhimurium* strains. The first examination of Ag nanoparticles as an anti-mutagenic substance was conducted by Sarac et al. in which *Streptomyces griseorubens AU2* was used for biosynthesis [176]. These Ag nanoparticles showed strong anti-mutagenic activity toward *Salmonella typhimurium* strains TA100 and TA98. Similarly, biogenic Ag nanoparticles have been green-synthesized using curcumin from *Curcuma longa* (Zingiberaceae), which were examined as anti-mutagenic substances toward *Salmonella typhimurium* strains TA97a, TA98, TA100, and TA102 [177]. Being able to function as indirect mutagens to organisms that might share the same genetic vulnerabilities found in TA98 and TA100 strains, Cur-AgNPs were shown to be mutagenic in the presence (+S9) but not in the absence (S9) of metabolic activation. Several studies have been conducted to determine whether nanoparticles are mutagenic. Ag/AgCl nanoparticles have been biologically synthesized using bacteria similar to *Staphylococcus pasteuri* isolated from soil samples of the Zarshouran gold mine [178]. The mutagenic and antimutagenic activity of these biogenic Ag/AgCl nanoparticles were investigated. The results showed that the Ag/AgCl nanoparticles did not display a mutagenic effect and inhibited mutagenicity with a percentage of 99% which enhanced their use as an anti-mutagenic agent. Additionally, copper nanoparticles were synthesized using *Prunus mahaleb L*. extract for the first time and their biological activities were investigated [179]. The results of the Ames test showed that these biogenic copper nanoparticles were not mutagenic and had high anti-mutagenic activity of more than 40%. A recent study also showed that green-synthesized silver and zinc nanoparticles using *Eucalyptus camaldulensis gum* extract were non-mutagenic and non-toxic [180].

**Figure 9:**
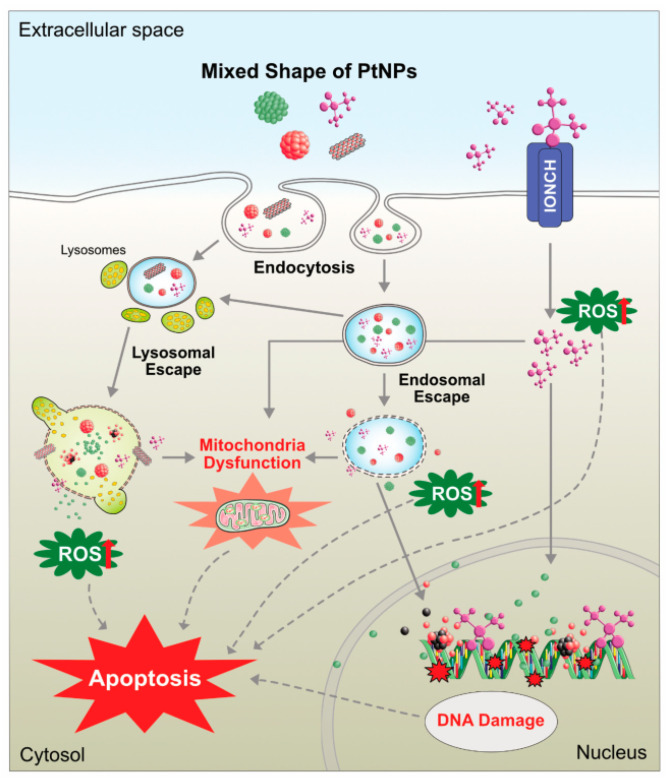
The possible mechanism of platinum nanoparticle-induced cytotoxicity in cancer cell lines. Copied from MDPI [186].

A cellular process called autophagy allows the cell to degrade its cytoplasmic components, including organelles and unfolded proteins, under certain stressful circumstances. Autophagy is induced in human cells when they are exposed to nanoparticles, which is associated with reactive oxygen species (ROS) and oxidative stress. Complex cellular processes are brought about by autophagic nanoparticles. Nanoparticles frequently cause pathogenic responses in cells. The autophagic reaction is governed by nanoparticle size and concentration. By reducing or impeding enzyme activity and interfering with lysosomal integrity and the vesicle trafficking cytoskeleton, NP accumulation in autophagosomes can disrupt autophagic flow. As a result, the risk of developing cancer and other diseases may increase and autophagy may be blocked. Several nanoparticles, such as iron oxide, titanium dioxide, alumina, and zinc oxide nanoparticles, can cause an autophagic response, which can prevent cell death [180,181,182,183,184]. According to a reported study on Ag nanoparticles, cells that have been exposed to too much ROS as a result of being treated with silver nanoparticles may exhibit impaired autophagic function. As a result, damaged organelles may accumulate and cause cells to experience oxidative stress. The ability of nanoparticles from various sources to influence the development of autophagosomes in treated cells has been investigated. High intracellular ROS concentrations in cancer cells subject the cells to greater oxidative stress, which can lead to cell death. ROS prefers to target cancer cells over healthy ones. The possibility of using autophagy to produce cell death as a cure for cancer and other neurodegenerative illnesses has made this topic more popular [185]. The treatment of various malignancies using green-synthesized nanoparticles has gained attention in recent years due to their ability to produce cytotoxic agents. However, these powerful agents can kill both malignant and healthy cells, making the safety of employing these materials a primary concern. The development of NPs as an anti-cancer medicine is an aim of researchers worldwide owing to their specific properties, such as being easy to produce, cost-effective, effective at the target site, a wide therapeutic index, biodegradability, and stability. The nanoparticle’s shape and size are significant factors because they determine the entry of particles into the cell and significantly affect the potentiation of cytotoxicity. For example, the shape of Pt nanoparticles can affect their cytotoxicity in cancer cell lines, as shown in Figure 9.

Other significant elements that affect the pharmacokinetics and pharmacodynamics of nanoparticles include the agglomeration/aggregation state, solubility, surface characteristics, attached functional groups, surface area, and surface charge. According to the literature, compared to gold, silver plant-meditated nanoparticles have stronger cytotoxic activity. The morphology and size of the particles have a major impact on cytotoxicity [187,188]. Metallic nanoparticles with smaller sizes have a higher chance of being cytotoxic in cell lines. Additionally, it has been demonstrated that nanoparticles may be less cytotoxic in normal cell lines, although this does not necessarily mean that they are less harmful. Another study found ZnO nanoparticles to be far less toxic to normal cells. The type of cell and the substance utilized both affect cytotoxicity. According to the study, ZnO nanoparticles were highly lethal to cancer cells. Recently, biosynthesized α-Bi_2_O_3_, Mn-doped, and Zn-doped Bi_2_O_3_ nanoparticles were synthesized using *Salvadora persica* extract as the green reactive, reducing, and capping agents with sizes of 46, 32, and 41 nm, respectively [189]. The results of the MTT assays confirmed the potent cytotoxic activity of the synthesized nanoparticles against breast cancer (MCF-7) and human umbilical vein endothelial (HUVEC) cells. These results demonstrated that nanomaterials were more biocompatible with healthy cells and more harmful to malignant cells [190]. Nevertheless, more investigation into this issue is necessary.

### 5.5. Antiviral and Antimicrobial Effect

More research is being done to enhance antimicrobial qualities as concerns increase about the developing resistance of microorganisms to antibiotics. Metallic NMs can effectively stop a variety of microorganism species, according to in vitro antimicrobial research. The particle size of the compound and the kind of material utilized to create the nanoparticles are the main two elements that govern the antimicrobial effectiveness of metallic NMs [191]. At the moment, utilizing NMs has the greatest chance of lowering microbial drug resistance. Nanoparticles can prevent antibiotic resistance and biofilm development via several approaches. NMs are skilled at combating drug resistance because they can activate a variety of processes that exhaust microorganisms by simultaneously promoting gene alterations to counter the mechanisms. However, it is unlikely that an organism would have several gene alterations in a single cell.

It is well known that Au nanoparticles have biocompatible and antimicrobial features. Au nanoparticles cannot function as an antimicrobial substance by themselves; they require the assistance of other biomolecules. To facilitate their bonding with other macromolecules, they can be cross-linked with gelatin, collagen, or chitosan [192]. By preventing the generation of virulence components, such as exopolysaccharides, and metabolic activity, such as surface hydrophobicity, which are crucial for bacterial-host cell interactions and biofilm architecture in microorganisms, respectively, Au NPs impair the biofilm development of *Proteus* spp. [193]. As reported in the literature, the antibacterial mechanisms of Au nanoparticles occur via clumping within the biofilm, biofilm assembly, flagella loss, and bacterial surface adhesion [194]. Additionally, Au nanoparticles can inhibit the growth of microbial cells via different mechanisms by impairing ATP synthase activity, which alters the membrane potential of the cell and causes cell death by collapsing the energy metabolism. For instance, synthesized Au nanoparticles with a size of 2 nm showed enhanced antibacterial properties [195]. Au nanoparticles can act as antifungal agents as they can affect and damage the membrane proteins via interaction with cell wall macromolecules [196]. This interaction causes cell damage due to changes in cell wall stability, resulting from the inhibition of cell wall β-glucan synthase [197]. Additionally, by increasing ROS, Au nanoparticles can exhibit antifungal action (for example, in *C. albicans*) [197]. *A. fumigatus*, *A. terreus*, *A. flavus*, *C. albicans*, and *C. tropicalis* are the most affected by Au nanoparticles as antifungal agents [198,199,200].

Another metallic nanoparticle, zinc oxide, has photo-oxidizing and photocatalytic activities that ensure its particular antibacterial and antifungal capabilities are safe in application. ZnO nanoparticles are efficient against microbes between a nanometer and micrometer in size. Because of its size, the particle enters the bacterial cell and disrupts its internal systems. Due to its large volume-to-surface area ratio and unique physiochemical properties, it has effective antibacterial effects [192]. Due to their effectiveness against both Gram-positive bacteria, such as *S. Aureus,* and Gram-negative bacteria, such as *K. aerogenes*, *E. coli*, and *P. Aeruginosa*, ZnO nanoparticles are considered efficient anti-microbial materials [201].

Iron (Fe) nanoparticles work effectively as anti-microbial substances. Studies have demonstrated that Fe nanoparticles synthesized from various source extracts have potential against various diseases [202,203,204,205]. Different extracts of Fe nanoparticles and their target bacterial strains have been investigated in several studies. Medical gadgets now contain iron-based nanosystems, which are effective at preventing the growth of microbial colonies. On catheter surfaces, essential oils with iron nanoparticles have been applied. It has been demonstrated that doing so reduces the main biofilm formation of *S. aureus* and *Klebsiella pneumonia*. However, they lose their effectiveness as biofilm formation progresses. Additionally, it has been demonstrated that coating wound dressing fibers with patchouli oil that contains iron nanoparticles reduces the growth of *S. aureus* biofilms [206].

**Figure 10:**
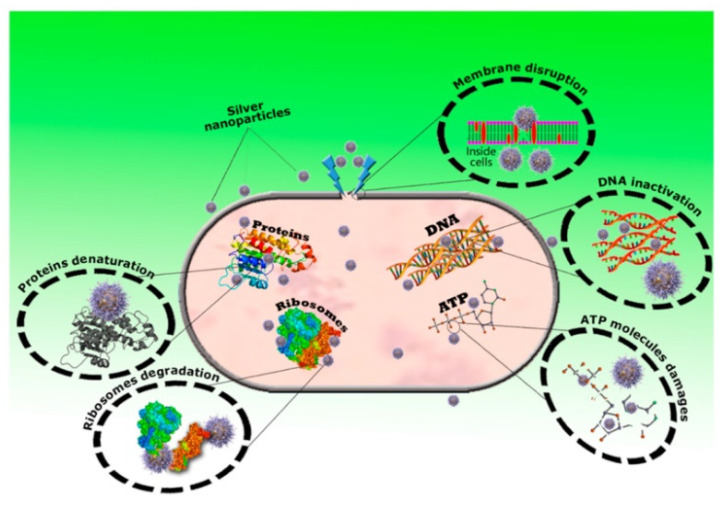
The possible antibacterial mechanism of green-synthesized Ag nanoparticles. Copied from MDPI [208].

Silver nanoparticles are one of the most sought-after nanoparticles in the medical field as an anti-bacterial agent. They work well as anti-fungal, anti-inflammatory, and antiviral treatments as well. *Escherichia coli* and other fecal coliforms are extremely susceptible to silver nanoparticles [207]. Figure 10 shows the possible mechanism of Ag nanoparticles as an antibacterial agent.

**Figure 11:**
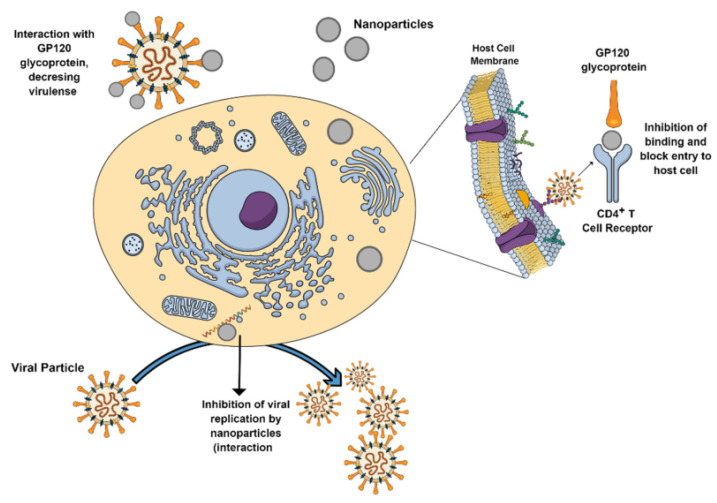
The possible mechanisms by which nanoparticles can act against different viruses. Copied from MDPI [210].

Silver nanoparticle size plays a significant role in antimicrobial resistance as well. Smaller silver nanoparticles have a greater surface area, which contributes to their improved ability to attach to various molecules. When silver nanoparticles penetrate a bacterial cell, they are transformed into silver ions, which harm the cell [192]. Additionally, silver nanoparticles can adhere to cell membranes, disrupting their permeability. The nanoparticles can help prevent the development of biofilms [209]. According to numerous studies, the antiviral effect of Ag nanoparticles may take place intracellularly by inhibiting viral reproduction or extracellularly by binding with viral protein (gp120) and preventing entry, which may differ depending on the kind of virus [86]. The possible mechanisms by which nanoparticles can act against viruses are shown in Figure 11.

However, the interaction between Ag nanoparticles and microbes remains unclear and requires more investigation. Ag nanoparticles are known to be an effective antiviral agent against several types of viruses, such as Human parainfluenza virus type 3, bovine Herpesvirus, Norovirus, Chikungunya virus, Dengue virus, Herpes simplex virus, Adenovirus, HIV, Influenza virus, and feline coronavirus (FCoV) [211,212,213,214,215,216,217,218,219,220,221]. Biogenic selenium, tellurium, cerium, and palladium nanoparticles have also demonstrated considerable antibacterial action in addition to the nanoparticles listed above. It is intriguing to note from the examples above that nanoparticles created utilizing microbe extracts are effective at killing other microbial species and increasing the effectiveness of current medications to combat antimicrobial resistance traits.

### 5.6. Drug Delivery Agents

Due to their non-toxic nature, targeted delivery, controlled drug release properties, bioavailability, biocompatibility, and stability, green-synthesized nanoparticles are significant candidates as drug delivery substances over conventional materials. Liposomes, micelles, emulsions, water-soluble polymers, and nanosphere materials are examples of such nanosubstances [222]. The ability to encapsulate a specific medicine and release it conditionally at the illness site is required for drug carriers. Additionally, to ensure targeted drug administration at the location, delivery agents must be able to overcome cellular barriers and blood tissue for inter- and intracellular transport [223]. Nevertheless, it is important to evaluate their effectiveness in cancer cells and their danger to normal cells right away. The development of a nanoparticle drug delivery system can solve the problem of the blood-brain barrier, which blocks the effective delivery of drugs and leads to treatment failure of various drugs [224]. Mesoporous silica nanoparticles, zinc oxide nanoparticles, gold nanoparticles, silver nanoparticles, and others are examples of nanoparticles for drug delivery [225,226,227,228].

Synthetic polyethylene glycol (PEG) nanoparticles that include antibiotics are referred to as “PEGylation.” This improves the effectiveness and precision of drug delivery. PEG coatings increase the effectiveness of nanoparticle formation, enabling the medicine to target bacterial infections in the body [229]. Magnetic greigite Fe_3_S_4_ and/or magnetite Fe_3_O_4_ are known to be transformed by magnetotactic bacteria into bilayer membrane-bound structures known as magnetosomes, which can be employed to encapsulate and transport pharmaceuticals [230]. In TC-1 mouse models, anti-4-1BB agonistic magnetosomes from *Magnetospirillum gryphiswaldense* were employed as immunotherapy against cancer [231]. When tested on H22 tumor-bearing mice, bacterial magnetosomes loaded with doxorubicin demonstrated greater tumor suppression than doxorubicin alone [232]. Transferrin-functionalized biogenic gold nanoparticles may also be able to penetrate the blood-brain barrier and deliver medications to the brain [233]. For anti-tumor drug delivery, taxol was coupled to gold nanoparticles synthesized with *Humicola* spp. fungi [234].

In addition, Fe_3_O_4_ nanoparticles are becoming more and more common in drug delivery systems because of their exceptional magnetic capabilities, often known as superparamagnetism. Fe_3_O_4_ nanoparticles must possess a few crucial characteristics that cannot be ignored if they are to be used as a drug delivery vehicle. Several different medication classes, including anti-inflammatory and anti-cancer drugs, can be combined with Fe_3_O_4_ nanoparticles. One experiment showed that indomethacin, a non-steroidal anti-inflammatory medicine with low water solubility, could be coupled with Fe_3_O_4_ nanoparticles and added to electrospun nanofiber composites made of two cellulose derivatives. The findings demonstrated that the composite nanofiber exhibited superparamagnetism at room temperature and that the presence of Fe_3_O_4_ nanoparticles in the nanofiber had little impact on the drug release mechanism, which was shown to be primarily regulated by the characteristics of the polymeric carrier matrix [235]. By using amino group coordination, Yan Zhang et al. created a nanocomposite with a core of Fe_3_O_4_ and polymeric inner shell filled with PEG and folate groups that contained cisplatin [236]. To prevent and treat cancer, curcumin, a naturally occurring polyphenolic hydrophobic molecule obtained from the rhizomes of *Curcuma longa*, has been added to Fe_3_O_4_ nanoparticles. The hydrophobic interface between the components of the surfactant-stabilized nanocarriers is where this herbal ingredient is supposed to stay bound [237]. Moreover, berberine has shown anti-tumorigenic capabilities; however, its hydrophobic characteristics made it poorly bioavailable at tumor locations. To overcome this constraint, this chemical was coupled with Fe_3_O_4_ nanoparticles and sanazole, and the resulting nanosystem decreased the size of hypoxic tumors in mice [238]. Thus, the excellent efficiency of Fe_3_O_4_ nanoparticles as drug delivery agents arises from their magnetic properties. Humans are administered medication-loaded Fe_3_O_4_ nanoparticles by parenteral drug delivery. The drug-loaded Fe_3_O_4_ nanoparticles can be injected into blood capillaries and located at the desired site (cancer cells/tumor) with the help of an external magnetic field. This can boost the effectiveness of treating cancer cells without hurting nearby healthy cells, thus allowing the medicine to concentrate and be released at the desired location. Subsequently, nanoparticles utilized in drug delivery systems should be safely designed by investigating their non-toxicity, biodegradability, and sterility, since they are used to release specific drugs performing certain functions in cells.

### 5.7. Bioimaging and Imaging Agents

Because it is affordable and efficient, fluorescence imaging is frequently employed in preclinical research. Over the past ten years, fluorescence photography has evolved to enable us to visualize biological processes at various scales, including the molecular, organellar, cellular, or even whole organism scale. Nanoparticle usage in bioimaging has recently grown in popularity. Nanoparticles are strong contenders for use in bioimaging due to their excellent optical properties, capacity as carriers for a variety of therapeutic and sensing components, and adjustable surfaces [239]. For thermal imaging of cancer tumors, several nanomaterials, including Pd, Au, Pt, Ag, and iron oxides, are utilized. Au nanoparticles exhibit luminous properties and have a high longitudinal relaxivity value. In a fascinating study, antibodies specific to the cell surface of liver cancer were coupled to AuNPs produced with *Candida albicans*. To distinguish liver cancer cells from healthy cells, the nanoparticles could specifically bind to the surface antigen found on liver cancer cells when utilized to probe those cells. Therefore, these nanoparticles could be used as a diagnostic tool or as a delivery system for anti-cancer medications [240]. Another often utilized nanoparticle is ZnO, which emits near-UV light and is crucial for excitonic blue luminescence, resulting in a green glow [241]. Due to their excellent hydrophilicity, optical transparency, and biocompatibility, fluorescent silica nanoparticles are also utilized in bioimaging [242]. Compared to Figure 6a, where aberrant or malignant cells are prominently displayed by luminous cells, optically active nanoparticles (Figure 6b) make it more difficult to detect the existence of such cells [243]. Therefore, green-synthesized nanoparticles play an important role in bioimaging and imaging processes. Table 4 summarizes the different applications of green-synthesized nanomaterials.

## 6. Limitations of Green-Synthesized Nanoparticles

Although green synthesis of NMs has a lot of potential, it is hindered by many factors, such as product quality control, synthesis circumstances, material choice, and product application. Adoption in industrial manufacturing and widespread use of green-synthesized NMs is hampered by these factors. The first limitation is the quality of green-synthesized NMs. There is high variability in the shape and size of NMs, which are produced using different extracts and microbes, and some have insufficient properties. According to recent reports, there is significant variability in particle size, making green technology unsuitable for large-scale manufacturing or making it difficult to control particle size during production. Some products have flaws in the way they are made, meaning that green-synthesized NMs differ widely in shape, including non-spherical shape, non-crystalline (amorphous), spherical, hexagonal, and irregular clusters. The practical benefits of the created products will be severely constrained by these differences, their use will be limited, and mass production will be challenging. Additionally, the low yield and conversion rate of green-synthesized nanoparticles is another limitation. In some cases, the conversion rate of metallic ions to nanoparticles does not exceed 50% and the metal content in samples may not exceed 9%. Because only a tiny number of nanoparticles can be produced from a high concentration of metal ions, this phenomenon indicates the poor conversion rate and low usage rate of metal ions. As a result, the economic gain is minimal. The second limitation is the selection of materials. There are numerous plant materials for the green synthesis of NMs, and numerous researchers have investigated locally and abundantly available plants. These investigations show that it is possible to utilize local plants to the fullest extent possible, but it is challenging to realize large-scale worldwide production of green-synthesized NMs. Time restrictions also prevent raw materials from being used in actual manufacture, such as differences in the flowering season of some plants from one country to another. Additionally, some plants require many years to grow and others grow at high altitudes. Moreover, some raw materials are secondary products that require additional processing, which increases the technology’s cost and complexity before they can be employed in the eco-friendly synthesis of NMs. Thus, it is necessary to confirm the applicability, economic viability, and cost-effectiveness of these materials. The third limitation is the application of green-synthesized NMs. For example, the use of these materials in removing pollutants is not efficient in all cases and did not exceed 10% in some experiments after a long processing time. Moreover, these green-synthesized NMs are ineffective in removing hazardous metal mixtures. To use green-manufactured NMs for this kind of practical application, the efficiency must be increased. The fourth limitation is the synthesis process, of which many concerns arise, such as the use of some industrial reagents, long reaction time, and excessive energy consumption. In some cases, the synthesis of green nanoparticles requires high temperatures that may exceed 800 °C. Because of this, some green synthesis processes need very high temperatures and lengthy synthesis times, which use a lot of energy and could be harmful to the environment. Even though green raw materials are employed, the method may not strictly adhere to the idea of “green synthesis.” In a different scenario, plant extracts must be stored until they are required. This process requires very low temperatures, which consumes a large amount of energy in the use of freezers. Therefore, it is wise to create nanoscale metals at normal temperature, which not only uses less energy but also simplifies the synthesis process. Additionally, the ideal reaction time should be quick because it affects both manufacturing efficiency and cost. Numerous studies, however, indicated the requirement for a longer reaction time. Further, a lengthy extraction procedure is likewise inappropriate. For example, some extracts required several days to be ready for use. Lack of knowledge of the mechanism underlying NP biosynthesis and difficulty in obtaining precise chemical reactions to describe the synthesis process are significant barriers to green biosynthesis. Indeed, it remains challenging to understand the precise reaction processes involved in the biosynthesis of nanoparticles. In the overall assessment of the green synthesis of nanoparticles, many challenging limitations must be overcome in the future.

## 7. Future Perspectives

Green synthesis (microbial synthesis) of NMs is a field that is continually evolving, with applications in many different industries to create cutting-edge, well-researched, and environmentally friendly solutions to different world problems. To completely optimize and capitalize on the advantages of biogenic nanoparticles, several areas need more research and development. The efficiency of various natural resources for the green synthesis of NMs, such as waste materials, microorganisms, or several biopolymers, has not been fully studied because the green synthesis of NMs is an extremely new field of research. It is important to increase the use of biological synthesis, particularly as it provides significant advantages that physical and chemical synthesis cannot offer. Nevertheless, it is difficult to fully utilize biological agents for NM synthesis since the fundamental mechanisms of the biosynthesis processes are poorly understood. Because of this, biosynthesis processes are variable and unexpected, even when producing the same substance. Synthesis might need a lot of time and money because it can be involved and complex. Therefore, it is necessary to employ probable synthesis techniques based on accurate and hypothetically predicted synthesis pathways. To achieve this, generally accepted theories and rules must be developed, which calls for the continued development of sophisticated and precise nano-investigation and monitoring techniques to investigate the kinetics of the processes. It is also necessary to have full knowledge of the molecular mechanisms, chemical agents, and biological elements included in the microbial production of NMs, as well as the ability to identify and separate the substances and comprehend the composition of the reducing and capping factors. The lab step remains essentially where green synthesis is most effective. Due to batch-to-batch variations, laboratory-controlled biosynthesis is currently unable to synthesize significant amounts of uniform NMs. As a result, eco-friendly, effective, reliable, robust, and continuous flow-based synthesis techniques are urgently required. For the manufacturing and use of NMs on an industrial scale, more thorough investigations and techno-economic feasibility studies are needed. Additionally, studies that examine the optimization of parameters impacting the growth of the microorganisms and the synthesis of NMs must be conducted to avoid long synthesis times using microorganisms. The majority of commercially accessible NMs have not been designed with ecological sustainability and safety as their top priorities. Furthermore, the negative impacts of NMs are not sufficiently understood. To reduce the dangers of toxicity, the application of NMs requires inspection and additional insight into their mode of action and movement. It is strongly advised to carefully review the entire raw material life cycle, from manufacture to discharge. Risk management throughout production, processing, preservation, and discharge also requires more focus.

## 8. Conclusions

Microbe-produced NMs show promise for a variety of therapeutic and biomedical applications because of their precisely calibrated biocompatible diameters and distinctive characteristics. Biosynthesis methods are also beneficial because NMs are frequently covered with biomolecule entities or a lipid layer that provide stability and physiological solubility, which are crucial for medical applications and are a limitation of other synthesis techniques. For large-scale use, biogenic NMs present a few problems that must be solved. Their application on a large scale has been constrained to this point by their lack of monodispersity, batch-to-batch variability, poor production rates, and labor-intensive production procedures. In response to this challenge, the current review addressed various environmentally friendly NM synthesis techniques, such as phytosynthesis, microbial-mediated synthesis, and enzyme-based synthesis, demonstrating the potential of various organisms and biomolecules to be used as nanofactories during green synthesis. In contrast to traditional approaches, this review has shown that these nanofactories are quite ecologically friendly, cost-effective, and simple to scale up. For example, the phytosynthesis technique showed that the fruits of plants, flowers, peels, leaves, shoots, and roots can be used as nanofactories containing various components that help during synthesis by acting as capping and reducing agents as well as contributing to the therapeutic effect of the substances. The advantages of using plant extracts are increased by the fact that they are simple to use and easily accessible. Additionally, NMs can be produced using a variety of microorganisms, including actinomycetes, algae, yeasts, fungi, and bacteria. Studies have shown that the microbial synthesis technique extracellularly or intracellularly incorporates events occurring during synthesis, such as trapping, bio-reduction, and capping. These biogenic NMs have shown wide applications in different fields, including the food industry, water treatment, and textile industry, as well as biomedical applications. Hence, this review discussed the use of different biogenic NMs in various applications. Moreover, the review discussed a plan to advance the field of green synthesis. Therefore, this review is a good start for scientists to begin their work in the green synthesis of NMs from where others have left off.

## Figures and Tables

**Table 1:** Comparison of different approaches for the synthesis of nanomaterials.

Item	Biological Synthesis	Chemical Synthesis (Bottom-Up)	Physical Synthesis (Top-Down)
Disadvantages	-Very expensive -Need for aseptic cultivation conditions	-Stabilizing and reducing agents are toxic materials and solvents. -Requires for high energy -Produces secondary harmful products.	-Requires high energy -Very expensive
Advantages	-Simple and easy method -Uses eco-friendly materials -Cost-effective methods	-Possibility of large scale production.	-Associated with enhanced purity -Size is controlled. -Shape is uniform. -Crystallinity is controlled.

**Table 2:** Characterization techniques used to identify the properties of NMs.

Characterization Technique	Obtained Properties
Microbial colony viability, in vivo, in vitro cell viability	Biological properties
High-performance liquid chromatography (HPLC)	Content
Mass spectrometry (MS)	Surface properties, structure, composition, molecular weight
Zeta potential	Stability of surface charge
Microscopy, Double photon correlation spectroscopy	Optical properties
Electrokinetics (Such as cyclic voltammetry studies)	Electrical properties
Dynamic light scattering (DLS)	Hydrodynamic size distribution
Differential scanning calorimetry (DSC)	Possible interactions of polymers and drugs, physicochemical state
Atomic force microscopy (AFM) Dynamic light scattering Electron microscopy (transmission/scanning)	Surface properties, aggregation, structure, shape, size, and size distribution
X-ray diffraction, Brunauer–Emmett–Teller (BET)	Surface and topographical properties
Transmission electron microscopy (TEM)	Aggregation, shape heterogeneity, size, and size distribution
Field emission scanning electron microscopy (FESEM) Scanning tunneling microscopy (STM) Scanning electron microscopy (SEM)	Aggregation, shape, size, and size distribution
Near-field scanning optical microscopy (NSOM)	Size and shape
X-ray photoelectron spectroscopy (XPS)	Chemical and elemental composition at the surface
Fourier transform infrared spectroscopy (FT-IR) Electron dispersive X-ray spectroscopy UV-visible spectroscopy	Chemical properties
Nuclear magnetic resonance (NMR)	Conformational change, purity, composition, structure
Infrared spectroscopy (IR) Raman spectroscopy Surface enhanced Raman spectroscopy (SERS)	Functional group analysis, Conformation and structure of conjugates

**Table 3:** Microbial synthesis of NMs using different types of organisms.

Microorganisms	Green-Synthesized Nanoparticles	Size	Applications	Ref.
Actinomycetes	ZnO nanoparticles	11.57 nm	Antibacterials and anti-biofilms against pathogenic microbes	[59]
	Au nanoparticles	45 nm	Antimicrobial and anticancer activity	[62]
	CuO nanoparticles	20 nm	Antibacterial and anticancer activity toward lung cancer cells	[67]
Algae	Ti nanoparticles	50 nm	Antistatic and anticancer activities	[72]
	Ag nanoparticles	13–31 nm	Antibacterial activities	[74]
	Ag and Au nanoparticles	50 and 20 nm	Antimicrobial activities	[75]
	SnO_2_ nanoparticles	32.2 nm	Photodegradation of dyes, antibacterial activity, Antioxidant activity, and cytotoxicity	[76]
	Ag nanoparticles	25–60 nm	Antibacterial activity	[77]
Phytosynthesis	Y_2_O_3_ nanoparticles	20–45 nm	Photodegradation of dyes, antibacterial Activity, cytotoxicity, and drug release	[80]
	Ag and Au nanoparticles	20.67 and 16.76 nm	Antioxidant activity, and antimicrobial activity	[81]
	Ag nanoparticles	20 nm	Antibacterial activity, preventing the coagulation of blood samples, and catalytic reduction of dyes	[82]
Viruses	Au and Ag nanoparticles	5–12 and 5–20 nm	Bio-semiconductors	[93]
	Fe_2_O_3_, PbS, SiO_2_, and CdS nanoparticles	22, 30, 24, and 5 nm	-	[95]
Fungi	Au nanoparticles	5–200 nm for extracellular synthesis and 10–25 nm for intracellular synthesis	-	[101]
	titania and silica nanoparticles	10.2 and 9.8 nm	-	[104]
	Se nanoparticles	55 nm	Antioxidant and antimicrobial activities	[105]
	ZnO nanoparticles	2–6 nm	-	[106]
	Fe_3_O_4_ nanoparticles	20–40 nm	Removal of Cr(VI) ions from water	[107]
	Carbon quantum dots	5.5–7.5 nm	Sensing of tetracyclines and bioimaging of cancer cells	[108]
Yeast	Se^0^ nanoparticles	50–250 nm	anti-candida and anti-oxidant activities	[110]
	Se nanoparticles	71.14 nm	Antioxidant activities, stimulated humoral immune potential, and trace element feed additive	[111]
	CdS nanoparticles	1–1.5 nm	Fabrication of an ideal diode	[112]
	Ag nanoparticles	2–5 nm	-	[113]
	Au nanoparticles and nanoplates	7.5–27 nm	-	[114]
	PbS quantum dots	2–5 nm	Semiconductors	[116]
Bacteria	Ag nanoparticles	20-40 nm	Antibacterial activities	[119]
	Ag nanoparticles	20 nm	-	[123]
	ZnO nanoflowers	3.8 nm	Agricultural applications	[124]
	Spherical ZnO nanoparticles	250 nm to 1 μm	Photocatalytic degradation of dyes	[126]
	ZnO nanoparticles	100–120 nm	In textile fabrics to enhance UV-blocking, self-cleaning and antibacterial properties, photocatalytic activity, and anticancer activities	[128]
	Pd^0^ nanoparticles	4–20 nm	Catalytic activity in dehalogenation reaction	[130]
	Ag nanoparticles	42–92 nm	-	[135]
	Au nanoparticles	20.93 nm	Antioxidant activity and an antiproliferative effect against cancer cells	[136]

**Table 4:** Different applications of green synthesized nanomaterials.

Applications	Green-Synthesized Nanoparticles	Activity	Refs.
Food industry	Ag, ZnO, and Fe_2_O_3_ nanoparticles	Antimicrobial and colorant agents in the food industry	[244]
	TiO_2_ nanoparticles	Used in food packaging and as additives in the food industry	[153]
	SiO_2_ nanoparticles	Used as additives in the food industry	[245]
Water treatment	Fe_2_O_3_ nanoparticles	Have great potential as photocatalysts for degradation of organic dyes	[158,159]
	MgO nanoparticles	Have great potential as photocatalysts for degradation of methylene blue dye	[161,162]
	TiO_2_ nanoparticles	Have great potential as photocatalysts for degradation of toxic heavy metals and tannery wastewater	[160,161]
Textile industry	Ag, Au, and ZnO nanoparticles	Provide textile fabrics with antistatic, antimicrobial, resistance to wrinkles, UV protection, flame-retarding ability, hydrophobicity, and self-cleaning properties	[128,166,167,168,169,170]
	CuO nanoparticles	Used for the synthesis of antimicrobial cotton fabrics and degradation of organic dyes used in textile colorization	[171,172]
	Silica nanoparticles	Popular for treating textile products and improving the hydrophobic qualities of the fabric surfaces	[246]
Mutagenicity	Ag nanoparticle	Are non-mutagenic and showed anti-mutagenic activities	[175,176,177]
	Ag/AgCl nanoparticles	Are non-mutagenic and showed anti-mutagenic activities	[178]
	Cu nanoparticles	Are non-mutagenic and have high anti-mutagenic activities	[179]
	Ag and Zn nanoparticles	Are non-mutagenic and non-toxic	[180]
Autophagy	Ag nanoparticles	Used to promote autophagosome buildup in cancer cells	[185]
	TiO_2_ and Mn nanoparticles	Can cause cellular autophagy	[247]
Cytotoxicity	Ag and Au nanoparticles	Cytotoxic agents that inhibit various types of cancers	[187,188]
	ZnO nanoparticles	Noticeably less lethal to normal cells and cytotoxic to cancer cells	[190]
Antiviral and antimicrobial agents	Au nanoparticle	Have antimicrobial, antifungal, and bio-compatible properties	[194,195,196,197,198,199,200]
	ZnO nanoparticles	Have efficient antibacterial and antifungal properties	[192,201]
	Fe nanoparticles	Have a notable anti-microbial properties	[202,203,204,205]
	Ag nanoparticles	Used as an antiviral, anti-bacterial, anti-fungal, and anti-inflammatory agents	[86,209,211,212,213,214,215,216,217,218,219,220,221]
Drug delivery	Mesoporous silica, ZnO, Au, and Ag nanoparticles	Efficient drug delivery agents	[225,226,227,228]
	polyethylene glycol nanoparticles	Increase the precision of drug delivery to target bacterial infections in the body	[229]
	Fe_3_S_4_ and Fe_3_O_4_ nanoparticles	Encapsulate and transport pharmaceuticals, immunotherapy against cancer, and tumor suppression agents	[230,231,232]
	Au nanoparticles	Delivery of medications to brain and anti-tumor drug delivery	[233,234]
	Fe_3_O_4_ nanoparticles	Used as a drug delivery vehicles for different medication classes	[235,236,237,238]
Bioimaging and imaging	ZnO nanoparticles	Used in bioimaging systems	[241]
	Silica, Fe_3_O_4_, Ag, Pt, Au, and Pd nanoparticles	Fluorescent silica particles are used in bioimaging and other nanoparticles are used in cancer tumor thermal imaging	[242]

## Data Availability

Not applicable.
